# Development of Partial Ontogenic Resistance to Powdery Mildew in Hop Cones and Its Management Implications

**DOI:** 10.1371/journal.pone.0120987

**Published:** 2015-03-26

**Authors:** Megan C. Twomey, Sierra N. Wolfenbarger, Joanna L. Woods, David H. Gent

**Affiliations:** 1 Oregon State University, Department of Botany and Plant Pathology, Corvallis, Oregon, United States of America; 2 US Department of Agriculture-Agricultural Research Service, Forage Seed and Cereal Research Unit, Corvallis, Oregon, United States of America; Virginia Tech, UNITED STATES

## Abstract

Knowledge of processes leading to crop damage is central to devising rational approaches to disease management. Multiple experiments established that infection of hop cones by *Podosphaera macularis* was most severe if inoculation occurred within 15 to 21 days after bloom. This period of infection was associated with the most pronounced reductions in alpha acids, cone color, and accelerated maturation of cones. Susceptibility of cones to powdery mildew decreased progressively after the transition from bloom to cone development, although complete immunity to the disease failed to develop. Maturation of cone tissues was associated with multiple significant affects on the pathogen manifested as reduced germination of conidia, diminished frequency of penetration of bracts, lengthening of the latent period, and decreased sporulation. Cones challenged with *P*. *macularis* in juvenile developmental stages also led to greater frequency of colonization by a complex of saprophytic, secondary fungi. Since no developmental stage of cones was immune to powdery mildew, the incidence of powdery mildew continued to increase over time and exceeded 86% by late summer. In field experiments with a moderately susceptible cultivar, the incidence of cones with powdery mildew was statistically similar when fungicide applications were made season-long or targeted only to the juvenile stages of cone development. These studies establish that partial ontogenic resistance develops in hop cones and may influence multiple phases of the infection process and pathogen reproduction. The results further reinforce the concept that the efficacy of a fungicide program may depend largely on timing of a small number of sprays during a relatively brief period of cone development. However in practice, targeting fungicide and other management tactics to periods of enhanced juvenile susceptibility may be complicated by a high degree of asynchrony in cone development and other factors that are situation-dependent.

## Introduction

Powdery mildews are problematic in several agricultural systems because many of the production practices that maximize yield involve high planting density, late-maturing cultivars, and generous fertilizer and irrigation inputs, all of which favor this pathogen group. Powdery mildews may damage yield through reductions in photosynthetic capacity and efficiency, accelerated senescence, and direct damage to the harvested tissue or fruit [1,2]. An important aspect of disease management is a basic understanding of the potential for and epidemiological understanding of crop damage and its determinants. Such an understanding provides strategic guidance to deploy appropriate control measures.

All of the above production practices converge in hop (*Humulus lupulus*) and make *Podosphaera macularis*, the causal agent of hop powdery mildew, one of the most destructive and costly diseases of this crop worldwide [3,4]. Hop is a dioecious perennial plant that produces annual bines [5]. The main commercial value of the plant resides in the female strobiles (cones), which are produced mainly for brewing, either directly or after extraction of the bittering acids [6]. Crop damage from powdery mildew is thought to be associated primarily or entirely with infection of cones. Depending on the end use of cones, total yield of alpha-acids may be most important for cultivars intended for extraction, although a combination of bittering acids, aroma, and subjective crop quality factors may be important when hops are used directly in brewing. Hop cones destined for direct use in brewing may be affected substantially by degraded crop quality because the crop value is largely determined by organoleptic factors such as appearance and smell, and metrics of brewing value [4,5,7]. Development of powdery mildew on cones is reported to contribute undesirable flavors during brewing [3,4,5]. Infection of cones by *P*. *macularis* also affects quality through discoloring (browning), whereas yield reductions are due to loss of developing cones. Infection of hops by *P*. *macularis* is also associated with indirect yield damage owing to shattering of bracts and bracteoles during mechanical harvesting operations [7]. Shattering at harvest is influenced by dry matter content of the cones, a correlate of cone maturity, with increasing dry matter content at harvest associated with progressively larger yield damage. The presence of powdery mildew may increase these indirect losses because the incidence of cones with powdery mildew is proportional to their dry matter content [7]. In this sense, damage from powdery mildew on cones can be described as both an "assimilate sapper" and "senescence accelerator" [8].

In other hosts, development of powdery mildew can provide an infection court for colonization by secondary, saprophytic organisms or pathogens. For instance, in grape even diffuse powdery mildew colonies on clusters are associated with development of a suite of microorganisms that can adversely affect wine quality [9]. It is unknown if a similar process occurs on hop cones infected with *P*. *macularis*. Several weak pathogens are known to colonize hop cones and may cause cosmetic damage in rare instances. Alternaria cone disorder, caused by the fungus *Alternaria alternata*, is one such disease. *A*. *alternata* is commonly recovered from discolored cones worldwide [10,11]. In Pacific Northwestern U.S.A., cone discoloration and associated premature senescence of hop cones has been increasingly attributed to Alternaria cone disorder since powdery mildew became endemic in the late 1990s [12].

The impact of powdery mildew on yield and quality of hop can be mitigated by strategic management actions [7,13,14], and therefore knowledge of the epidemiology of the disease process is of utmost importance. It has long been recognized that management actions during bloom are important determinants of the severity of powdery mildew at harvest [5]. More recent studies found that yield damage from powdery mildew is influenced by fungicide applications during and just after bloom. Termination of fungicide applications before bloom or the transition from bloom to cone development, the so-called stage II of cone development [15], was associated with a 20% reduction in yield of alpha-acids [7]. However, there was relatively minor yield damage when fungicide applications were terminated after stage II of cone development, provided plants were harvested before dry matter content was not unduly high. Further, a meta-analysis of fungicide trial data found that highly efficacious fungicides such as quinoxyfen applied during a three week period centered on stage II of cone development nearly halved the incidence of cones with powdery mildew at harvest compared to less efficacious fungicides applied during this period [16]. Previous work by Seigner et al. [17] utilizing detached tissues also reported, qualitatively, some attenuation of disease susceptibility with cone maturation.

Collectively, these previous studies suggest a period of juvenile susceptibility to powdery mildew and the development of some level of ontogenic resistance in hop cones. Decreasing susceptibility of tissues to pathogens as they mature is a common phenomenon in powdery mildew diseases and has been described in several pathosystems (e.g. [18,19,20,21]). Understanding if, when, and to what degree ontogenic resistance exists may enable management tactics to be reconciled to the most critical stages of host susceptibility. This may allow for a reduction in the number or intensity of fungicide applications necessary to successfully manage the disease.

In this research, we sought to characterize and quantify the level of ontogenic resistance to powdery mildew in hop cones. Research is presented from greenhouse assays, field experiments, and commercial hop yards which describe the phenology of powdery mildew susceptibility, its consequences on cone quality factors and components of the infection process, and the potential implications for disease management.

## Materials and Methods

### Inoculum production and inoculation

Leaves bearing colonies of *P*. *macularis* were collected from multiple commercial hop yards in Oregon, all within Marion County. No specific permits or permissions were necessary for these activities or locations. However, isolates were maintained according to conditions outlined in permit P526P-11-02217 issued by the US Department of Agriculture Animal and Plant Health Inspection Service. Neither collection of the powdery mildew samples, nor the other studies described below, involved endangered or protected species.

Chains of conidia from these colonies were transferred to detached leaves of hop cvs. ‘Symphony’ and ‘Pacific Gem’ that were previously surface disinfected by spraying with 70% ethanol for 30 seconds, rinsed with sterile deionized water, and air dried. These cultivars were selected for their high degree of susceptibility to all known strains of powdery mildew. Single spore isolations were not conducted to preserve a population of *P*. *macularis* representative of that found in natural environments. Inoculated leaves were maintained in double stacked Petri dishes with the petiole inserted in water in the lower dish and leaves in the upper dish. After inoculation, leaves were incubated for two weeks at 18°C under a 14-hour photoperiod. Subsequent maintenance and increase of inoculum was through successive transfers onto cv. ‘Symphony’ in a growth chamber maintained at 16°C with a 14 h photoperiod.

### Greenhouse experiments to measure ontogenic resistance

During 2009 and 2010, plants of cv. ‘Symphony’ were grown in 4 liter pots in MetroMix 840PC potting soil (SunGro Horticulture, Agawam, Massachusetts) in a greenhouse isolated from other hop plants and free of powdery mildew. Plants were watered regularly and supplied with Sunshine Technigro 16-17-17 Plus fertilizer with micronutrients (Sun Gro Horticulture) at each irrigation to promote vigorous growth. Shoots were trained onto 3 m strings to promote flowering. Greenhouse temperatures were maintained at 20 to 25°C with at least a 14-h photoperiod provided by supplemental artificial lighting.

From mid-July to early October, flowers or cones were removed from the plants every 7 to 10 days. Entire flowers or bracts and bracteoles dissected from developing cones were placed on sterile, moistened germination paper in Petri dishes. The tissue was inoculated with conidia of *P*. *macularis* utilizing a settling tower as described previously [22], the inoculum being derived and maintained as described above. A set of bract and bracteole tissues was placed on the germination paper with either the adaxial or abaxial surface facing upward to test for differential susceptibility to powdery mildew. In each inoculation at least 10 flowers, bracts, or bracteoles were inoculated per replication. Juvenile leaves of the same cultivar were included in all assays as a positive control to verify inoculum viability. Inoculated tissue was incubated in a growth chamber maintained at 18°C for 10 days, approximately 1.8 to 2 latent periods [23], before assessing the incidence of tissue bearing conidiophores of the fungus. Cone tissues were assessed beyond the point when cones are typically harvested to quantify susceptibility of the tissue over a broad range of maturity and to the point of cone senescence. The experiment was structured as a completely randomized design with at least three replications per inoculation date.

The incidence of powdery mildew was plotted in relationship to days since bloom and the relationship indicated a curvilinear functional form. Nonlinear, least-squares curve fitting was conducted using several models implemented in SigmaPlot version 11.0 (Systat Software, San Jose, California) that described a logistic or exponential decrease between two variables. The best fitting model that provided a reasonable description of the data was selected based on the pseudo-*R* ^2^, standard error of parameter estimates, and residual diagnostics.

### Field experiments to measure ontogenic resistance

During each year from 2011 to 2013, plants of cv. ‘Symphony’ were propagated from soft wood cuttings in a greenhouse free of powdery mildew and maintained as described previously. Eventually, plants were potted into 20 liter pots and trained onto 2 m tall bamboo stakes.

Between mid-May and early June of each year the plants were deployed to an Oregon State University research farm near Corvallis (44°38'2.54" N 123°11'24.30" W) isolated from other hop plants to minimize the likelihood of developing powdery mildew from outside sources. The plants were irrigated by drip irrigation up to four hours a day (as needed) and fertilized with approximately 200 ml of a solution of Sunshine Technigro 16-17-17 Plus every two weeks. The plants were treated with paraffinic oil (1% solution of JMS Stylet Oil, JMS Flower Farms, Vero Beach, Florida), potassium bicarbonate (6.8 g per liter of Kaligreen, Otsuka AgriTechno, Tokyo, Japan) and/or cymoxanil (1.2 g per liter of Curzate 60DF, DuPont Crop Protection, Wilmington, Delaware) every one to two weeks to minimize the development of powdery mildew and downy mildew. These applications ceased at least 14 days prior to a given inoculation, as described below. Plants at the isolation site were grown until lateral branches were produced which bore cones in the desired developmental stage. Fifty flowers or cones per plant in the desired developmental stage were inspected with the aid of a 30× hand lens to ensure the cones were free of powdery mildew. Cones that were not in the desired developmental stage were marked at the top of the rachis with red fabric paint and were not assessed further but remained on the plant. The plants were then transferred to the Oregon State University Lewis-Brown Horticulture Farm (44°33'9.36"N, 123°12'56.98"W) for inoculation. Inoculations were conducted on each of five dates during 2011, 2012, and 2013 at cone developmental stages corresponding to the stages I, II, III, IV, and V [15]. In brief, stage I corresponded to the late stages of bloom. Stage II was a transition stage from bloom to cone development wherein stigmas begin to abscise and bract and bracteole development become conspicuous. In stage III, cone volume and mass began to increase linearly, stigmas were fully senesced and abscised, and bracteoles were approximately half the length of the bracts. In stage IV, development of bracteoles proceededsuch that they were slightly open and no longer closely enclosed the ovary. Stage V was full maturity, wherein bracteoles were fully elongated and the ovary was inconspicuous.

Inoculations in stage I to V occurred, respectively, on 30 June, 7 July, 15 July, 21 July, and 5 August during 2011; 6 July, 19 July, 25 July, 9 August and 14 August in 2012; and 11 July, 18 July, 1 August, 15 August, and 22 August in 2013. Mean daily temperature during the experiments ranged from 15.1 to 21.4°C in 2011, 15.7 to 27.2°C in 2012, and 16.9 to 24.6°C in 2013. Inoculum of *P*. *macularis* was grown and maintained as stated above and prepared by washing conidia from several heavily infected cv. 'Symphony' leaves with ultra pure water (Nanopure with organic-free cartridge kit, Aqua Solutions, Deer Park, Texas) with Tween 20 (0.05% vol/vol). The inoculum titer was adjusted to 100,000 conidia per ml with the aid of a hemacytometer and promptly sprayed onto the desired cones using a Preval airless paint sprayer (Chicago Aerosol, Bridgeview, Illinois) until just before run-off. The inoculated plants were placed in a shaded area for approximately 18 hours to promote infection and then transferred to an outdoor pot-in-pot system without shade at the same farm in a randomized complete block design with two to eight plants per replication. The plants were irrigated for approximately four hours daily by drip irrigation and maintained at this site until cone harvest. As a confirmation of infectivity of the inoculum, greenhouse-produced plants of the same cultivar were inoculated with the same spore suspension and maintained at 18°C with a 14 h photoperiod for 10 days.

### Cone harvest and quality determination

Inoculated cones were harvested from each of the plants 11 to 13 days after inoculation of cones in stage V. The incidence of cones with powdery mildew was assessed under a stereomicroscope at 10 to 50× magnification. Disease severity was assessed on 10 arbitrarily-selected cones per plant using an 0 to 8 ordinal scale, with categories defined as follows: a rating of 0 indicated no signs of *P*. *macularis* observed under 50× magnification; 1 indicated few conidiophores visible under bracts and bracteoles at 50×; 2 indicated numerous conidiophores present under bracts and bracteoles but with the absence of distortion of the cone and external mycelia; 3 indicated fully elongated cones with no distortion due to powdery mildew, but with limited external mycelia and conidiophores visible by an unaided eye; 4 indicated fully elongated cones with distortion from powdery mildew on up to 25% of the cone area and mycelia externally visible on up to 25% of the cone; 5 indicated a cone elongated to greater than 75% of the length of an unaffected cone, 25 to 50% of the cone was externally colonized by mycelia, and most powdery mildew was found under bracts/bracteoles; 6 indicated an elongated cone 50 to 75% of the length of an unaffected cone, with distortion caused by powdery mildew on up to 50% of the cone, and externally visible mycelia on 50 to 70% of the cone; 7 indicated cones 25 to 50% the length of unaffected cones, with distortion on 50 to 75% of the cone, and with macroscopically visible external mycelia on 70 to 90% of the cone; and 8 indicated cones that were less than 50% the length of unaffected cones with more than 90% of the surface colonized by *P*. *macularis*.

**Fig 1:**
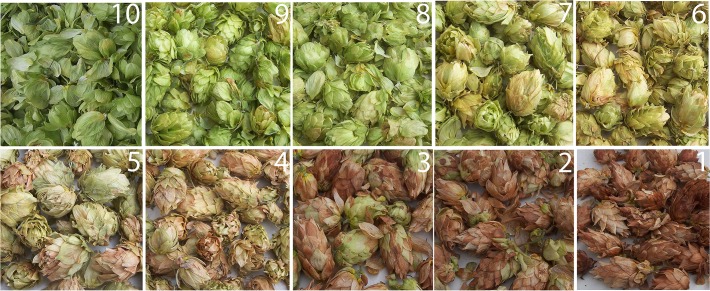
Cone color scale used for evaluation of color defects associated with powdery mildew.

A subsample of at least 75 cones was placed in paper bags and dried overnight in a dryer at 48°C to 8 to 10% moisture content. Dried cones were rated for color using a 1 to 10 ordinal rating scale adapted from a commercial rating system used in hop brokerage (Fig. 1)[7]. Bittering acids content and the hop storage index was determined by standard spectrophotometric methods [24]. A separate subsample of at least 25 cones from each plot was collected and dried for 48 to 72 hours at 60°C to determine percent dry matter using standard methods [24].

The various measurements and data collected were analyzed separately for each year using a mixed effect model with replication considered a random factor. Analyses were carried out using the GLIMMIX procedure in SAS version 9.4 (SAS Institute, Cary, North Carolina). A normal distribution was assumed for response variables except those measured on an ordinal scale. Ordinal ratings for cone color and disease severity were analyzed using a nonparametric analysis appropriate for designed experiments [25,26].

Additionally, data from the field experiments to characterize ontogenic resistance were analyzed jointly in a mixed effect model. The assorted response variables were modeled to be dependent on the timing of inoculation, expressed as days after bloom. Linear, quadratic, and cubic polynomial terms were investigated, and polynomial terms were selected that yielded the simplest model that adequately described the data. Year, block nested with year, and the interaction of days since bloom*year were considered random factors in the analysis. Analyses were conducted using the GLIMMIX procedure, assuming a normal distribution of the response variable. Because disease severity ratings were based on an ordinal scale with uneven spacing, a multinomial distribution was specified for the disease rating values and a cumulative logit link function was utilized. Although the cone color ratings were also based on an ordinal rating scale, a normal distribution was assumed because the color rating scale was based on approximately evenly space categories (i.e., percent discoloration) and could be considered roughly normally distributed over the range of values considered.

### Conidial germination and penetration comparison between Stage II and Stage IV cones

Experiments were conducted during July and August 2013 to quantify differences in germination, penetration, and microcolony development on cones of varying maturity. Cones in stage II or stage IV were collected from a set of four plants at the isolation site. Bracts were removed from cones and placed on moistened germination paper in Petri dishes. The tissue was inoculated with conidia of *P*. *macularis* utilizing a settling tower as described for the greenhouse experiments on both the adaxial or abaxial surfaces. A set of bracts from stage II and stage IV was prepared but not inoculated to serve as a control for potential pre-existing infection. Plates were incubated in a growth chamber at 18°C for 72 hours. After incubation, bracts were fixed in a 3:1 (vol/vol) ethanol/glacial acetic acid solution. Tissue was transferred to fresh solution as needed until completely cleared. Bracts were stained with Coomassie Brilliant Blue R-250 (0.12 g/L Coomassie (Sigma-Aldrich, St. Louis, Missouri) in an aqueous solution containing 50% (vol/vol) methanol and 10% (vol/vol) glacial acetic acid) for 5 to 10 seconds to stain the mycelium before rinsing with deionized water two to three times. Bracts were mounted on a microscope slide in 50% glycerol and viewed under 100× magnification using a compound light microscope. All conidia present on the tissue were classified as germinated or non-germinated. A conidium was considered germinated if a germ tube was present and elongated to at least half the length of the conidium. Amongst germinated conidia, 10 were selected arbitrarily from each bract evaluated and the proportion that had multiple or branching secondary hyphae was noted. Conidia with secondary branching were assumed to have penetrated the bract and are referred to as such hereafter. Hyphal measurements of all primary and secondary branching hyphae were made with an ocular micrometer. The experiment was replicated independently three times during July and August 2013, and structured as a randomized complete block design with blocking over time. Over all three replications of the experiment, 1543 conidia were assessed for germination, and of these measurements of hyphal development were made on 938.

### Latent period and conidia production over time

Studies were conducted in 2012 and 2013 to describe the latent period and production of conidia over time on cones in stages II and IV. Eight to 16 plants bearing cones in stages II and IV were inoculated 9 August 2012 and 15 August 2013 as described above for the field experiments to measure ontogenic resistance. During these studies, daily mean temperature ranged from 16.3 to 24.1°C in 2012 and 16.3 to 21.9°C in 2013. Beginning 5 days after inoculation, 3 cones were removed from each of at least two plants per replicate block and transported to a lab and examined with a stereomicroscope for the presence of conidiophores of *P*. *macularis*. Cones were similarly collected and assessed every 1 to 2 days thereafter to determine the duration of the latent period of the fungus. Additionally, conidia produced on cones were enumerated beginning 5 days after inoculation and every 3 to 4 days thereafter. Conidia were directly enumerated on cones until becoming too numerous to count. When too numerous to count directly, conidia were rinsed from cones with a Preval sprayer and the number in the rinsate estimated with the aid of a hemacytometer.

Measurements of conidia germination, penetration, and microcolony development were analyzed as a factorial structure in a mixed model with fixed factors for cone developmental stage (II and IV) and surface (abaxial and adaxial). Replication of the experiment over time was a random factor. Differences in latent period were expressed by fitting a 3-parameter logistic model to data for each stage as described for the greenhouse experiments. Parameter estimates from the models were used to calculate the point at which 50% of cones had sporulating colonies of *P*. *macularis*, defined as the LP_50_. Temporal differences in conidia production between cones in stage II and IV were expressed by fitting either a 3-parameter logistic model or a polynomial regression to the data.

### Colonization of epiphytic fungi related to timing of infection of *P*. *macularis* on cones

At the time of cone harvest in 2011 and 2012, 5 cones were arbitrarily selected from each plant prior to drying. Five bracts and bracteoles were selected from each cone and placed into a 10% bleach solution for 30 seconds followed by a 30 second rinse in sterile water. Once dry, 5 pieces of tissue approximately 2 to 4 mm^2^ in area were excised from each bract or bracteole from the margin of discolored areas, if present. In the instance of no apparent browning, 5 areas were arbitrarily selected for tissue excision. Tissue pieces were placed on ½ strength potato dextrose agar amended with 50 mg/ml streptomycin-sulfate (Sigma-Aldrich) and incubated at 18°C under 14 hours of light. Seven to 10 days later, plates were assessed under 10 to 25× magnification and fungal genera present were identified based on morphological characteristics. When identification was uncertain based on morphological characters, a culture was established from a hyphal tip transfer, and DNA was extracted using a MoBio Ultra-Clean Soil DNA isolation kit as per the manufacturer's instructions (MoBio Laboratories, Carlsbad, California). The ITS region was amplified using the primers ITS-1 and ITS-4 as described previously [27]. PCR products were sequenced bidirectionally at the Oregon State University Center for Genome Research and Biocomputing, and sequences were compared to other organisms available on the National Center for Biotechnology Information Genbank database for confirmation of genus. Isolation frequency of fungi from cones was analyzed as a randomized complete block design with developmental stage as fixed factor and block as random factor. Analyses were conducted using GLIMMIX assuming a binomial distribution of the response variables and logit link function.

### Impact of fungicide applications targeting the period of peak susceptibility of hop cones to powdery mildew

Since previous research [7,16] indicated that there was a critical period requiring fungicidal protection to suppress infection of hop cones by *P*. *macularis*, we examined whether fungicidal protection only during the juvenile stages of cone development resulted in similar disease control as a season long program of fungicide applications. Experiments were conducted on cv. 'Willamette', which is moderately susceptible to powdery mildew. Field sites were located in a 1 ha experimental hop yard near Corvallis, Oregon during 2011 and in two commercial hop yards near Salem, Oregon during 2010 and 2011. In the experimental plots, three treatments were evaluated: (i) a non-treated control that received no fungicide applications; (ii) a treatment that consisted of fungicide applications made every 10 to 14 days consisting of a rotation of trifloxystrobin (Flint 50W, Bayer CropScience, Research Triangle Park, North Carolina), spiroxamine (Accrue, Bayer CropScience), quinoxyfen (Quintec, Dow AgroSciences, Indianapolis, Indiana), and a pre-mix of boscalid + pyraclostrobin (Pristine, BASF, Research Triangle Park, North Carolina) as per standard production practices; and (iii) an application of quinoxyfen and boscalid + pyraclostrobin made 22 July and 4 August, respectively, during the predicted period of juvenile susceptibility. These two applications were made on the same dates in the plots that received the standard, season-long fungicide treatments. All fungicides were applied at the highest rates allowable on the manufacturers' label for hop in the U.S. Applications were made with an Eagle BP40 backpack sprayer (Eagle-1 Manufacturing, Monroe, Washington) in an application volume equivalent to 1870 liters/ha. A plot consisted of 8 hop plants, and plots were arranged in a randomized complete block design with four replications (32 plants total). Each plot was separated by one to two rows of untreated plants. The incidence of leaves with powdery mildew was assessed by inspecting 10 leaves from each plant in the plots (80 leaves per plot) every two weeks throughout the season. On 19 August, one to two lateral branches were removed from each of 4 plants in the center of each plot and transported to a laboratory. The cones were removed from the branches originating from a plot and mixed. From this composite sample, 50 to 100 flowers or cones were selected randomly and inspected for the presence or absence of powdery mildew with the aid of a stereomicroscope at 10 to 50× magnification.

In the commercial hop yards, two directly adjacent plots were established in each yard, each plot being five to seven rows wide by the length of the yard. The plots received either: (i) regular application of fungicides throughout the season as per the cooperating growers’ standard practices; or (ii) only two (2010) or three (2011) fungicide applications applied just before flowering and during the critical stages of cone susceptibility to powdery mildew. To minimize drift between plots, the cooperating growers turned nozzles off on half of the sprayer on the outer rows of the plot receiving the standard fungicide spray and disease measurements were made only in the center row of each plot. In 2010, the targeted application plots were treated with fungicides on 5 July with 1 kg/ha micronized sulfur (Microthiol Disperss, NuFarm, Morrisville, North Carolina) and on 26 July with 0.15 kg/ha quinoxyfen (Quintec). The grower’s standard applications included these two applications and additional fungicide applications on 15 April, 12 May, 24 May, 29 May, and 28 June consisting of 0.43 kg/ha copper ammonium complex (Copper-Count-N, Chemical Specialties, Charlotte, North Carolina), 35 g/ha trifloxystrobin (Flint) + 2.29kg/ha potassium bicarbonate (Kaligreen), 2.69 kg/ha micronized sulfur, 2.69 kg/ha micronized sulfur, and 4.59 kg/ha potassium bicarbonate + 0.14 kg/ha spiroxamine (Accrue), respectively. In 2011, the targeted application plot was treated three times. On 8 July, plots received 1.8 kg/ha micronized sulfur + 2.29 kg/ha potassium bicarbonate, and on 15 July and 27 July plots were treated with 0.18 kg/ha quinoxyfen + 2.29 kg/ha potassium bicarbonate. The standard applications included these three and additional applications of 1.8 kg/ha micronized sulfur made on 1 and 14 June, and 1.8 kg/ha micronized sulfur + 1.15 kg/ha potassium bicarbonate on 23 June. Fungicide applications were made with cooperating growers' standard spray equipment and commercial practices. The incidence of leaves with powdery mildew was measured using a modification of the methods presented in Gent et al. [28]. Ten leaves were assessed for the presence of powdery mildew on each of 100 plants in the middle row of each plot every two weeks. At harvest, the incidence of cones with powdery mildew was determined by collecting cones from each of 25 plants in the middle row of each plot from lateral branches at heights of approximately 2.7, 3.6, and 4.5m from the ground. The cones from each plant were bulked and 25 cones were selected and assessed for the incidence of powdery mildew.

Data from the fungicide timing experiment was analyzed in GLIMMIX as a linear mixed model with block as a random factor. The experiments in the commercial hop yards were not randomized across the yard due to logistical constraints of the cooperating growers. Therefore, difference in the incidence of cones with powdery mildew among plants between the two plots was assessed using unpaired *t*-tests (assuming equal variances) to test for differences among the incidence of cones with powdery mildew amongst the 25 plants sampled in each plot. Calculations were conducted in Excel 2007 (Microsoft, Redmond, Washington).

### Temporal development of powdery mildew and cone ontogeny

The temporal development of powdery mildew on cones was monitored during 2011 to 2013. Beginning at bloom and each week thereafter until early September, one to two lateral branches were removed from each of four plants in experimental plots of cv. 'Willamette' from heights of approximately 2.7, 3.6, and 4.5m from ground. Plots were as described for the non-treated control in the fungicide timing experiment. The branches were transported to a laboratory where the flowers and cones were removed from the branches originating from a plot and mixed (2011) or sorted by canopy height (2012 and 2013). In 2011, 50 to 100 flowers or cones from the composite sample per plot were selected and inspected for the presence of powdery mildew with the aid of a stereomicroscope. In 2012 and 2013, 20 flowers or cones were selected from each canopy height for disease assessments. The mean incidence of cones with powdery mildew was plotted against time to construct disease progress curves. Logistic models were fitted to the disease progress curves from the nontreated experiment plots for each year to estimate the rate parameter using standard methods [29]. Nonlinear regression was conducted using the NLIN procedure in SAS. In 2012 and 2013, the number of buds, flowers, and cones in the developmental stages of Kavalier et al. [15] were counted and categorized.

## Results

### Greenhouse experiments

**Fig 2:**
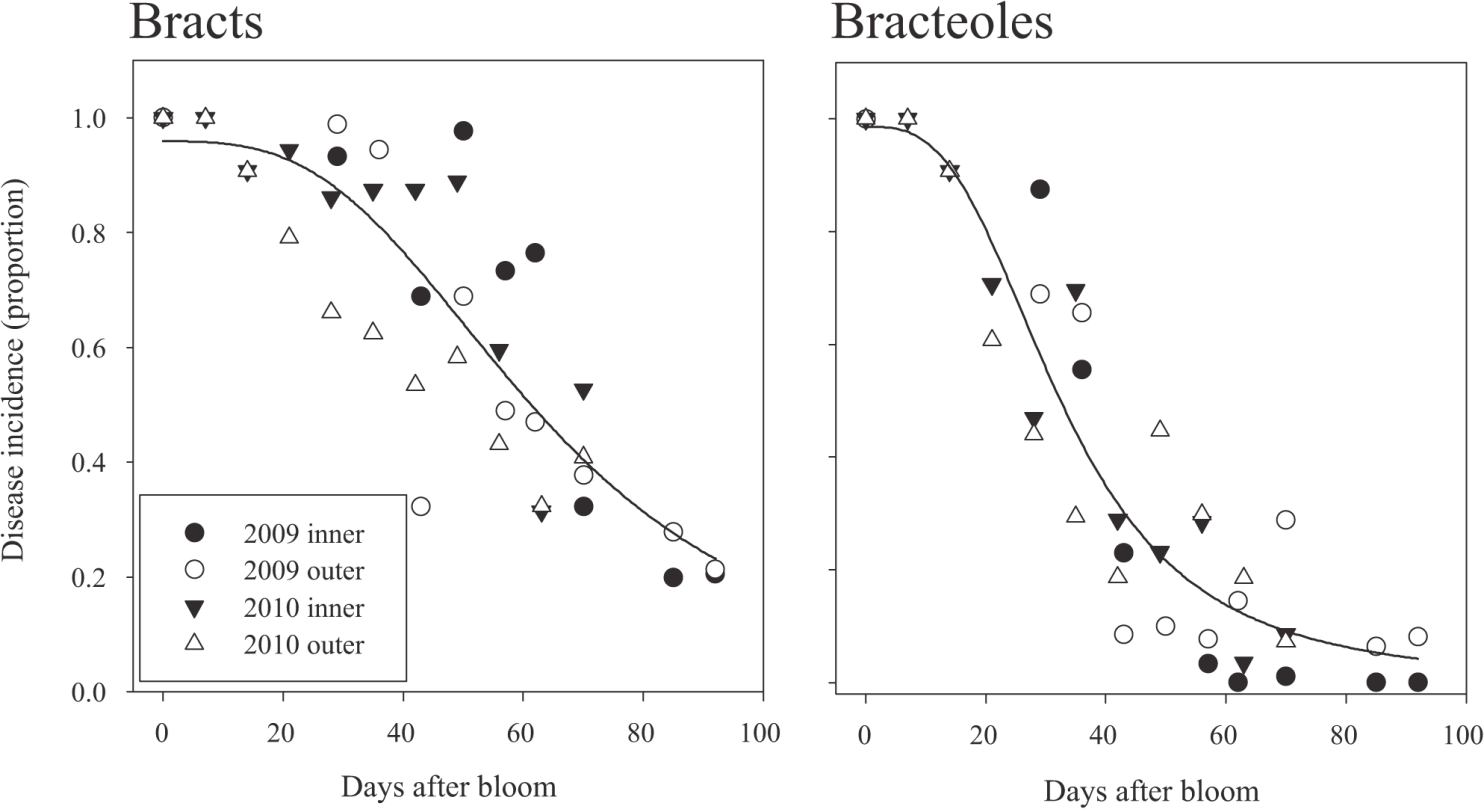
Powdery mildew susceptibility in greenhouse produced cone bracts and bracteoles of cv. ‘Symphony’. Comparisons in susceptibility are shown for 2009 (circles) and 2010 (triangles) between the inner tissue surfaces (solid) and outer tissue surfaces (open). The curves are three parameter logistic models (bract *R* ^2^ = 0.74, *P*<0.0001; bracteoles *R* ^2^ = 0.88, *P*<0.0001). See text for equations.

Leaves inoculated as positive controls became diseased in all experiments, verifying that inoculum was viable and conditions were permissible for infection. Flowers, bracts and bracteoles exhibited differences in their susceptibility to powdery mildew depending on developmental stage, with a clear pattern for greater juvenile susceptibility in bracts and bracteoles (Fig. 2). In both 2009 and 2010, inoculation of flowers resulted in disease incidence approaching 100%. The susceptibility of bracts decreased later in their development, near 30 to 40 days after bloom. The equation of a three parameter logistic model fit to the bract data was: Incidence of bracts with powdery mildew = 0.9590 / (1 + (days after bloom/63.0645)^3.0325^) Parameter estimates were significant at *P* < 0.0001 and the model coefficient of determination was 0.74.

The susceptibility of bracteoles decreased more rapidly and sooner than that of bracts, beginning near 20 to 30 days after bloom as flowers began the transition to cone development. The equation of a three parameter logistic model fit to the bracteole data was: Incidence of bracteoles with powdery mildew = 0.9864 / (1 + (days after bloom/32.7598)^3.0030^) Parameter estimates were significant at *P* < 0.0001 and the model coefficient of determination was 0.86. The inner surface of the bracts and bracteoles showed a general tendency for greater susceptibility to powdery mildew compared to the outer surfaces of these tissues.

### Field experiments to measure ontogenic resistance

**Fig 3:**
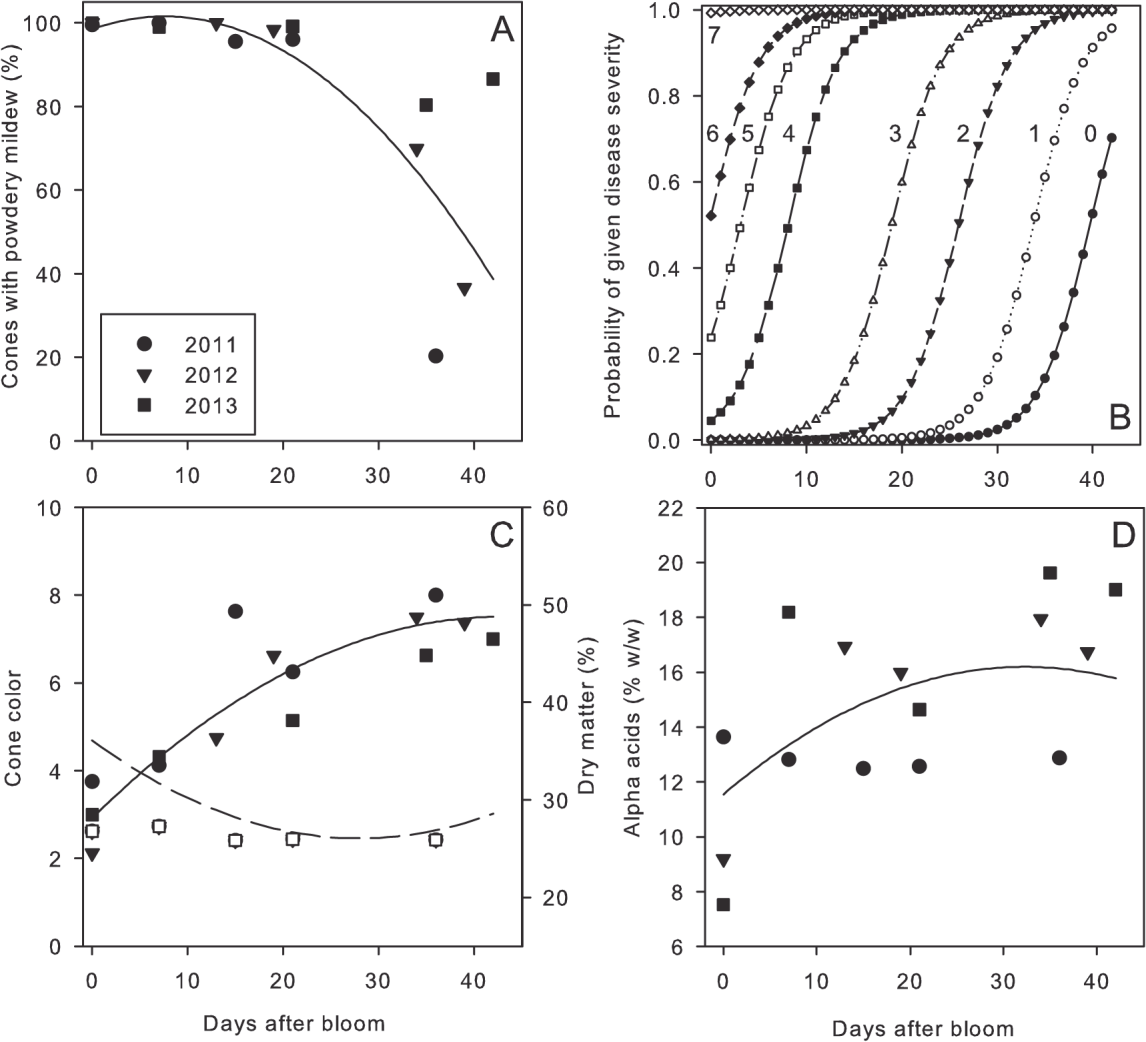
Association of inoculation timing in relation to powdery mildew development on cones and quality factors. **A and B.** Development of powdery mildew on cones in relation to the timing of inoculation with *Podosphaera macularis*. In **B,** the cumulative probability of disease severity being rated in a given disease class (ordinal scale of 0 to 8 as described in text) is expressed as a function of timing of inoculation expressed in days after bloom. Numerical values near each curve indicate disease severity class. **C.** Dry matter (dashed line, open symbols) and cone color (solid line, solid symbols) in relation to inoculation timing. **D.** Alpha acids content of cones in relation to inoculation timing. Lines in each figure are predicted values from a mixed model analysis; see text for equations and details.

Leaves inoculated as positive controls became diseased in all experiments. Some level of powdery mildew was detected on cones following inoculation at every developmental stage (Fig. 3A). The incidence of cones with powdery mildew approached 100% in all years on cones inoculated from bloom to 21 days after bloom (stage I to III). Incidence of cones with powdery mildew decreased in all years beyond approximately 20 days after bloom (Table 1). The severity of powdery mildew was greatest when cones were inoculated at stage I and II, and disease severity decreased progressively to nearly imperceptible levels by stage V (Fig. 3B). A mixed model equation describing the relationship between the incidence of cones with powdery mildew and timing of inoculation was: Disease incidence = 98.5157 + 0.8038(days since bloom) - 0.05302 (days since bloom)^2^ The parameter estimates for the quadratic term was significant (*P* < 0.0001) but not for the linear term (*P* = 0.264). For the analysis of disease severity, cones were more likely to receive a greater powdery mildew severity rating when inoculated near bloom as compared to later dates (*P* = 0.034).

**Table 1:** Disease development, quality, and bittering acids content of hop cones when inoculated with *Podosphaera macularis* in varying developmental stages^a^.

		Disease measurement^b^			Color quality factors^b^			Bittering acids (%)^c^		
Cone stage	Days after bloom	Incidence (%)	Severity (median)	RE (95% CI)	Color (median)	RE (95% CI)	Dry matter (%)	Alpha	Beta	HSI
2011										
I	0	99.6a	3	0.71 (0.68–0.74)	3.5	0.20 (0.13–0.36)	26.8	13.6	4.7	0.21
II	7	100.0a	4	0.89 (0.79–0.90)	4	0.24 (0.15–0.42)	27.3	12.8	4.5	0.23
III	15	95.5a	2	0.43 (0.38–0.47)	8	0.73 (0.58–0.82)	25.8	12.5	4.4	0.21
IV	21	96.0a	2	0.38 (0.33–0.43)	6	0.54 (0.38–0.69)	26.0	12.6	4.5	0.22
V	36	20.3b	0	0.10 (0.10–0.10)	8	0.80 (0.61–0.87)	25.9	12.9	4.5	0.21
	*P-value*	<0.0001		<0.0001		0.0005	0.107	0.421	0.731	0.264
2012										
I	0	100a	7	0.90 (0.90–0.90)	2	0.10 (0.10–0.10)	48.8a	9.2a	3.5a	0.23
II	13	100a	6	0.70 (0.70–0.70)	5	0.30 (0.30–0.30)	32.5b	16.9b	5.6b	0.24
III	19	98.4a	3.75	0.50 (0.50–0.50)	6	0.60 (0.40–0.76)	30.8b	16b	5.6b	0.25
IV	34	70b	2	0.30 (0.30–0.30)	7.5	0.78 (0.63–0.85)	29.0b	17.9b	5.5b	0.22
V	39	36.7c	0	0.10 (0.10–0.10)	7	0.73 (0.59–0.81)	29.5b	16.7b	5.2b	0.23
	*P-value*	<0.0001		<0.0001		0.006	<0.0001	0.0002	0.002	0.789
2013										
I	0	100a	6	0.92 (0.92–0.92)	3	0.10 (0.10–0.10)	34.5a	7.5a	2.4a	0.23
II	7	99a	4	0.63 (0.53–0.71)	4	0.33 (0.27–0.40)	28.1b	18.2c	5.7c	0.24
III	21	99a	4	0.58 (0.48–0.66)	5	0.47 (0.41–0.53)	27.0bc	14.6b	4.6b	0.23
IV	35	80.4c	1.5	0.26 (0.20–0.34)	7	0.78 (0.72–0.82)	24.6d	19.6c	6.1c	0.23
V	49	86.6b	1	0.14 (0.11–0.29)	7	0.83 (0.76–0.86)	25.0cd	19.0c	5.8c	0.23
	*P-value*	<0.0001		<0.0001		<0.0001	<0.0001	<0.0001	<0.0001	0.629

^a^ Cones were inoculated with a suspension of 10^5^ *P*. *macularis* conidia per ml on the dates indicated and measurements were taken at harvest during mid August to early September, depending on year. Treatments within a column and the same year followed by different letters are significantly different based upon a mixed model analysis with an *F*-protected least significant difference (α = 0.05).

^b^ Disease severity was assessed on a 0 to 8 ordinal scale, where 8 is the greatest possible disease severity as described in the text. Cone color was assessed on a 1 to 10 ordinal scale (Fig. 1). Ordinal data was analyzed using a nonparametric analysis and ANOVA-type statistic. Relative effect (RE) ranges from 0 to 1 and provides a measure of treatment effects for a given experiment [26]. Significant differences in relative effect are inferred by lack of overlap of the 95% confidence intervals, which are presented parenthetically.

^c^ Bittering acids and Hop Storage Index (HSI) were determined by the American Society of Brewing Chemists spectrophotometric method [24]. Hop Storage Index is a measure of relative loss of bittering acids. Increasing values indicate an increase in degradation of bittering acids.

**Fig 4:**
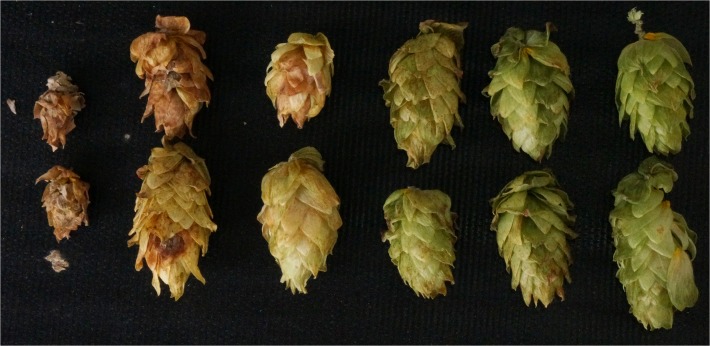
Hop cones at harvest when inoculated with *Podosphaera macularis* at progressively later stages of maturation. Left to right, cones were inoculated at stage I, II, III, IV, V or non-inoculated.

There was an inverse relationship between cone color and dry matter content, with the most severe reductions in color and greatest dry matter content resulting from inoculations at stage I or II (Fig. 3C and 4). The association between cone color or dry matter content in relation to timing of inoculation was given by the following mixed model equations: Cone color = 2.9251 + 0.2136(days since bloom) - 0.00249(days since bloom)^2^ and Dry matter content = 36.1084–0.7196(days since bloom) + 0.01287(days since bloom)^2^ All parameters estimates were significant (*P* ≤ 0.0009).

Similarly, alpha acids content of cones was related to timing of inoculation (Fig. 3D), with the most severe impacts on alpha acids when cones were inoculated at Stage I (Table 1). The mixed model equation was: Alpha acids content = 11.5475 + 0.2883(days since bloom) - 0.00447(days since bloom)^2^ Parameter estimates were significant (*P*≤ 0.0190).

### Conidial germination, penetration frequency, and microcolony development

Germination of conidia on bracts was influenced by cone maturity (*P* = 0.045), with a modest (10.9%) reduction in germination of conidia on bracts in stage IV versus stage II (Table 2). Germination rates were similar on abaxial and adaxial surfaces (*P* = 0.586). The fungus penetrated the host surface 50% less when inoculated onto cones in stage IV versus stage II (*P* = 0.010). Penetration was similarly reduced on the adaxial surface of bracts (*P* = 0.021). Microcolony development, as measured by total hyphal length, was independent of cone maturity (*P* = 0.482), but was reduced on the adaxial surface (*P* = 0.001). In none of the measurements did cone developmental stage interact with surface (*P* ≥ 0.365).

**Table 2:** Response of *Podosphaera macularis* when inoculated onto abaxial and adaxial surfaces of hop cone bracts in varying developmental stages^a^.

				Latent period (days)	
Factor	Germination (%)^b^	Penetration (%)	Hyphal development(μm)	2012	2013
**Development stage**					
II	56.6a	22.6a	327.2a	8.7	9.4
IV	45.7b	11.3b	306.1a	10.9	17.0
*P*-value	0.0448	0.0101	0.4820		
**Bract surface**					
Abaxial	52.8a	24.7a	341.8a		
Adaxial	52.9a	12.6b	287.1b		
*P*-value	0.5855	0.0214	0.0012		

^a^ A conidium was considered germinated if a germ tube was present and elongated to at least half the length of the conidium. Values presented are percentage of conidia evaluated that were germinated. Penetration was assumed if a conidium had secondary branching hyphae.

^b^ Means of variables are statistically similar if followed by the same letter according to a generalized linear mixed model (α = 0.05). Developmental stage × bract surface interactions were nonsignificant in all experiments (*P* ≥ 0.05). Germination, penetration, and hyphal development are means calculated from observations of 1543 conidia or 938 microcolonies evaluated over three replications of the experiments conducted during 2013. Latent period is the number of days after inoculation when 50% of hop cones bore sporulating colonies of *P*. *macularis*.

### Latent period and conidia production over time

**Fig 5:**
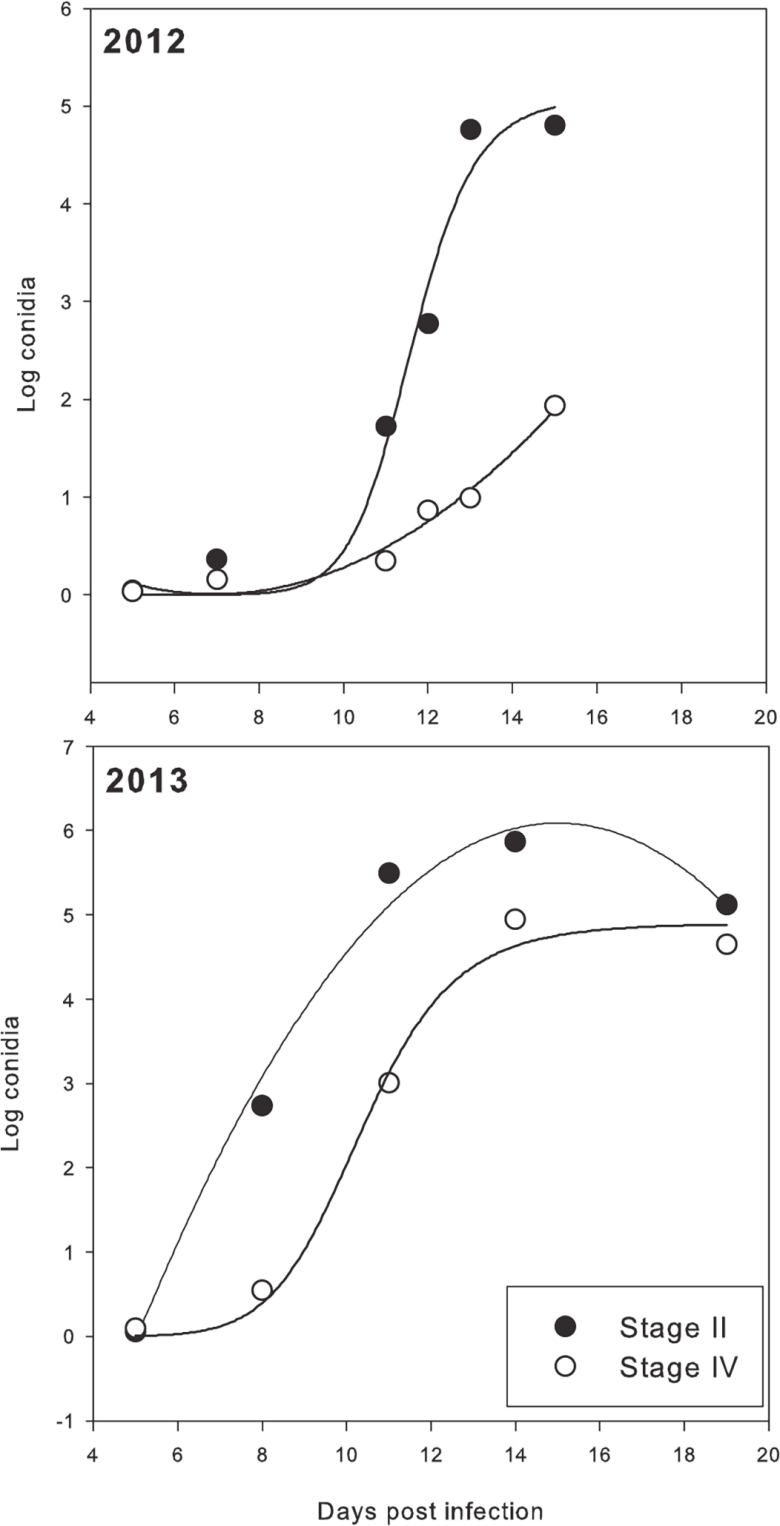
Conidia production on hop cones when inoculated in developmental stages II and IV. In 2012, curves are regression fits of a 3-parameter logistic model (stage II) and a quadratic regression model (stage IV). In 2013, curves are fits of a quadratic regression model (stage II) and a 3-parameter logistic model (stage IV). See text for equations.

The latent period of cones inoculated at stage IV was longer (2.2 to 7.6 days, depending on year) than cones inoculated at stage II (Table 2). Sporulation also was consistently reduced on more mature cones (Fig. 5). Equations describing conidia production in 2012 were: log(conidia) = 5.0812 / (1 + (days after inoculation/11.6169)^−15.5065^) for stage II and log(conidia) = 1.398–0.4015(days after inoculation) + 0.029(days after inoculation)^2^ for stage IV (Fig. 5).

Differences in conidia production between developmental stages generally were less pronounced in 2013 than 2012. The equations describing the 2013 data were: log(conidia) = −7.7327 +1.8427(days after inoculation) -0.06142(days after inoculation)^2^ for stage II and log(conidia) = 4.8999 / (1 + (days after infection/10.3616)^−9.4384^) for stage IV. Parameter estimates were significant in all models at *P* ≤ 0.0689 and the model coefficient of determination was not less than 0.96 in any model.

### Colonization of epiphytic fungi related to timing of infection of *P*. *macularis* on cones

In 2011, cones inoculated at stage I or stage II yielded a significantly greater isolation frequency of fungi in general, and *Alternaria* spp. and *Cladosporium* spp. specifically, than cones inoculated at stage III or later (Table 3). *Epicoccus* spp. also were isolated at a greater frequency in cones that were inoculated with *P*. *macularis* in juvenile cone stages as compared to cones inoculated at maturity (stage V). These trends were similar in 2012, and all fungi were isolated more frequently when cones were challenged with *P*. *macularis* in stage I or II. Of particular note, *Alternaria* spp. were recovered from 7.2% or less of isolations when powdery mildew was not present during the juvenile stages of cone development.

**Table 3:** Frequency of isolation of opportunistic and saprophytic fungi from hop cones when inoculated with *Podosphaera macularis* in varying developmental stages^a^.

		Frequency of isolation (%)			
Cone stage^b^	Date inoculated	***Alternaria*** spp.	***Epicoccus*** spp.	***Cladosporium*** spp.	Any culturable fungus
2011					
I	30 June	11.0a	9.5a	43.5a	53.5a
II	7 July	9.5a	9.3a	40.8a	50.5a
III	15 July	3.3b	5.0ab	25.8b	34.5b
IV	21 July	1.0b	4.0ab	16.0bc	23.0bc
V	8 Aug	2.0b	1.0b	12.0c	17.0c
	*P-value*	0.0004	0.0507	<0.0001	<0.0001
2012					
I	6 July	30.3a	68.5a	48.3a	95.0a
II	19 July	22.5a	22.3b	30.8b	61.0b
III	25 July	7.2b	15.2b	26.9bc	48.5c
IV	9 Aug	2.8c	12.0b	16.5d	30.0d
V	14 Aug	0.5c	18.5b	21.0cd	40.5c
	*P-value*	<0.0001	<0.0001	<0.0001	<0.0001

^a^ Cones were inoculated with a suspension of 10^5^ *P*. *macularis* conidia per ml on the dates indicated. Isolations from cones were made at harvest (16 August 2011 and 26 August 2012) onto half-strength potato dextrose agar. Isolation frequency was determined from 5 sections of necrotic tissue collected from 5 cones collected arbitrarily from each replicate plot (25 cone pieces per replication). Within a year, means within a column followed by different letters are significantly different based upon a mixed model analysis with an *F*-protected least significant difference (α = 0.05).

^b^ Cone developmental stages as described in Kavalier et al. [15]. Stage I is bloom and cones mature progressively in later stages.

### Impact of fungicide applications targeting the period of peak susceptibility of hop cones to powdery mildew

**Fig 6:**
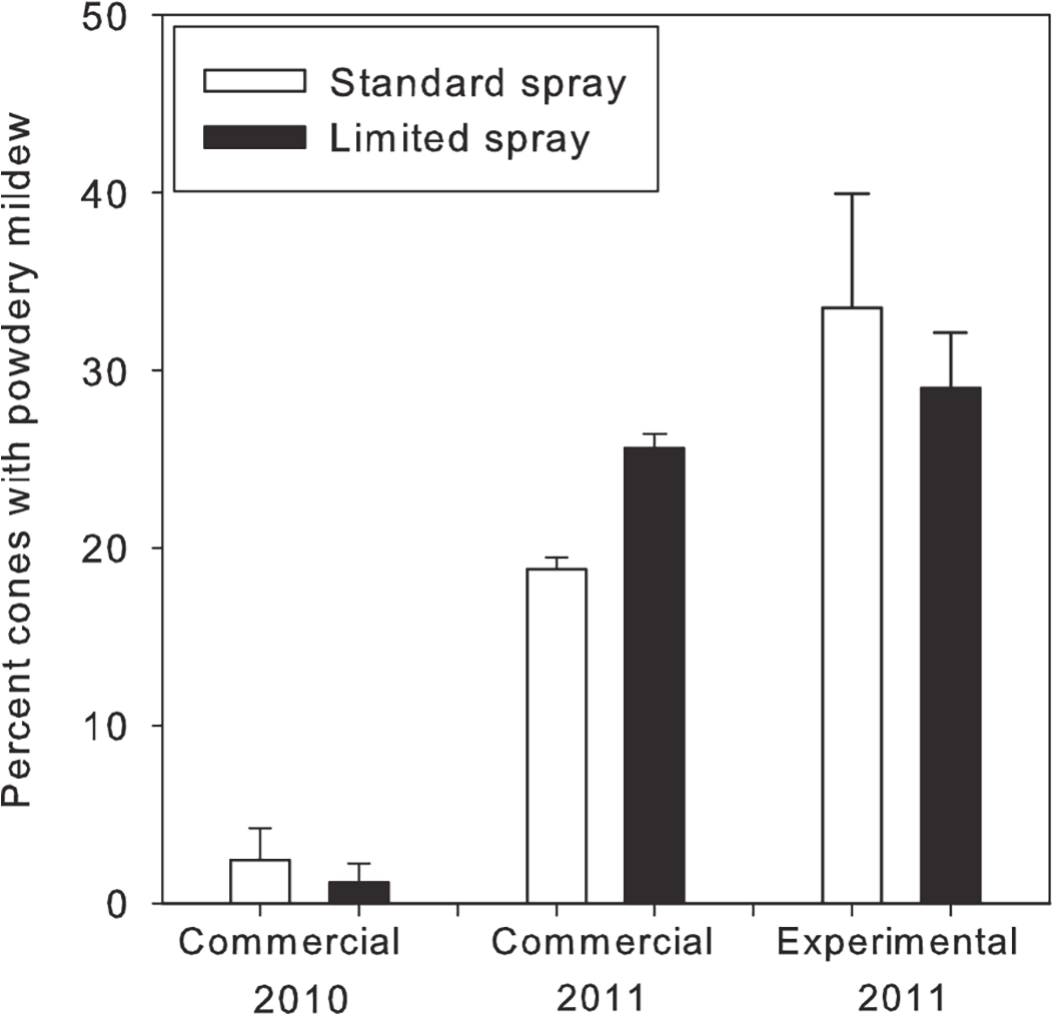
Comparison between standard and limited fungicide application programs on powdery mildew control. Standard season long fungicide application programs (open bars) are compared to two (2010 commercial yard and 2011 experimental plots) or three (2011 commercial yard) fungicide applications (solid bars) made during juvenile stages of cone development. Differences in disease incidence were not different according to *t*-tests (commercial yards) or mixed model analysis (experimental plots). Data is from cv. 'Willamette' in Oregon, USA. Bars indicate mean ± standard errors.

The incidence of cones with powdery mildew was statistically similar when fungicide applications were made for the duration of the season or targeted only to the juvenile stages of cone development (Fig. 6). In the commercial hop yard trials in 2010, plots that received full season fungicide applications developed powdery mildew on 2.4% of cones compared to 1.2% in plots that received only two fungicide applications (one-sided *t*-test *P* = 0.08). Incidence of leaves with powdery mildew was maximal on 23 June at 29.7% in plots that received the full season treatment and 26% in the plots with limited applications. In 2011, plots that received the full season spray program had 18.8% of cones with powdery mildew at harvest compared to 25.6% in plots that received three fungicide applications (one-sided *t*-test *P* = 0.08). The incidence of leaves with powdery mildew was greatest on 23 June at 25.8% with full season treatment and 31.7% for limited applications. In experimental plot trials in 2011, the incidence of cones with powdery mildew was 33.5% in plots that received full season fungicide applications compared to 29.0% in plots that received only two fungicide applications. Both the full season and limited spray treatments had significantly less powdery mildew than the nontreated (80% disease incidence; *P* < 0.0001). Leaf incidence was maximal on 28 July and was 4.7% in nontreated plots, 4.7% in plots that received full season fungicide applications, and 10.9% in plots that received two fungicide applications.

### Temporal development of powdery mildew and cone ontogeny

**Fig 7:**
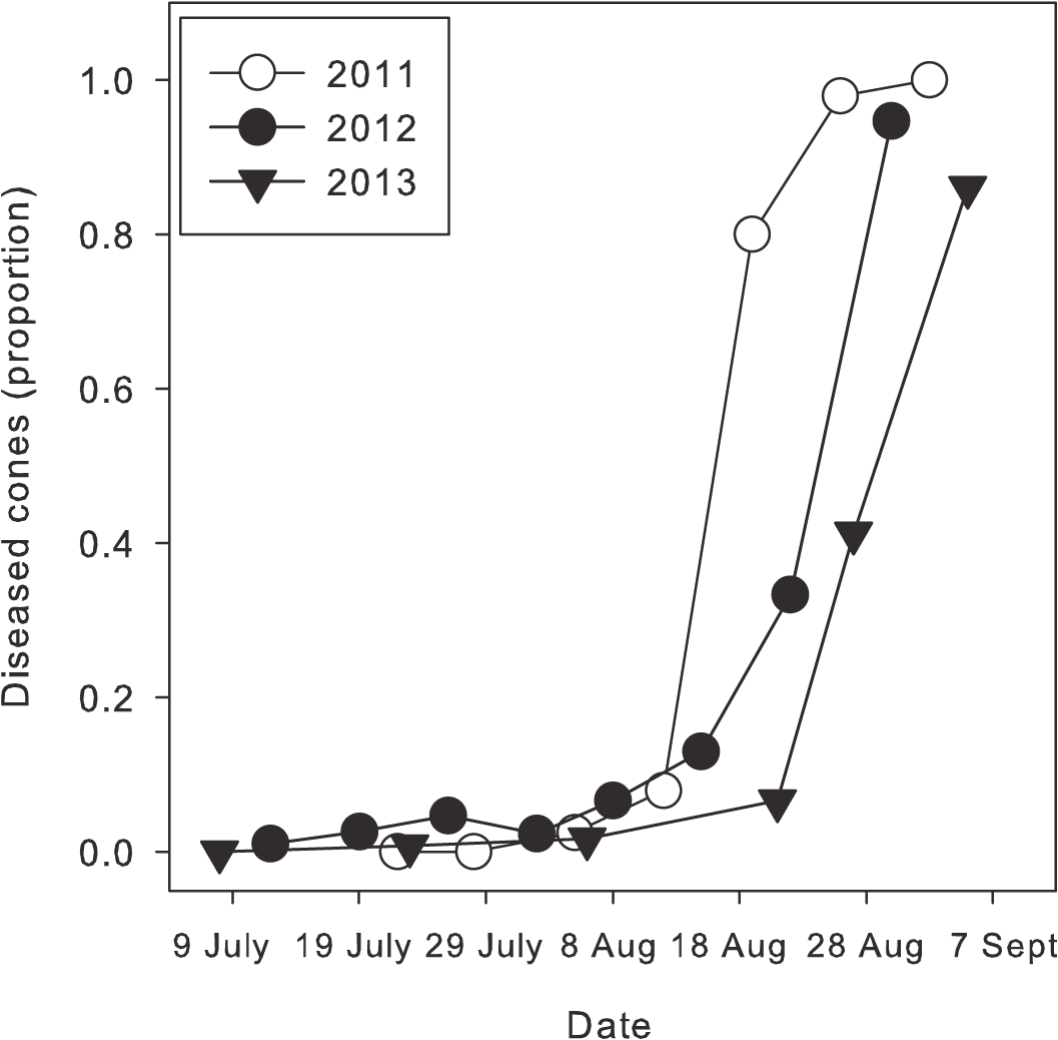
Development of powdery mildew on flowers and cones. Incidence of hop cones with powdery mildew in non-treated plots of cv. 'Willamette' during 2011, 2012, and 2013.

In the absence of fungicide applications, the incidence of cones with powdery mildew increased logistically over time in all years of the study (Fig. 7). On the last sampling date 100, 94, and 86% of cones had powdery mildew in 2011, 2012, and 2013, respectively. The rate of disease increase varied among years, though, with the rate parameter for the logistic model being 0.52 in 2011, 0.36 in 2012, and 0.28 in 2013.

**Fig 8:**
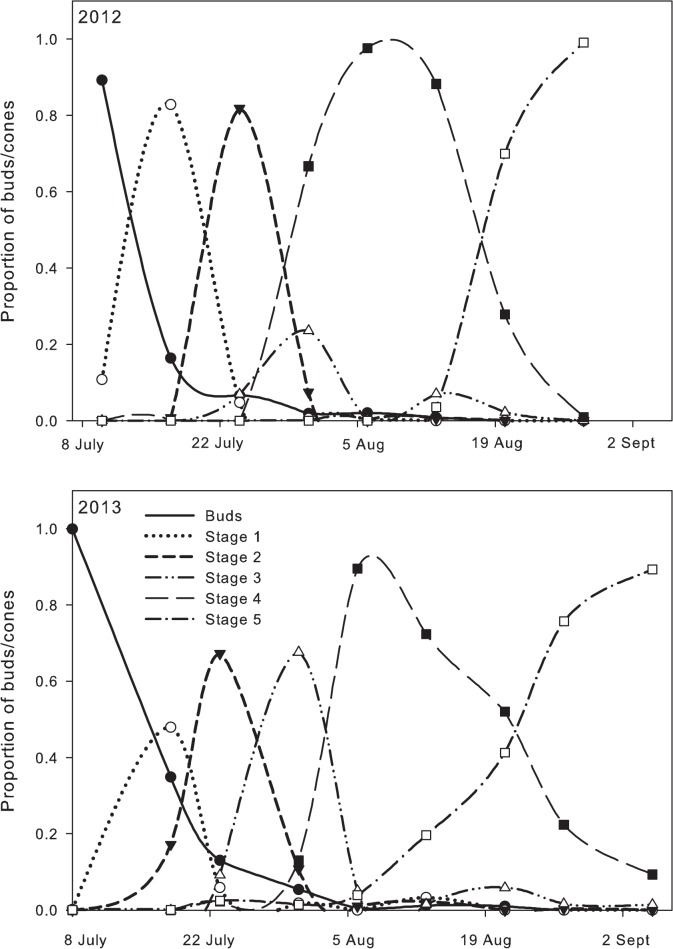
Temporal development of buds and hop cones during 2012 and 2013 on cv. 'Willamette'. Symbols are observed (measured) data and the lines are cubic spline interpolations fit to the data.

In both 2012 and 2013 there was a substantial overlap of stages in cone maturity (Fig. 8). Cones in stages I, II, or III were found over a period of 25 days in 2012 and 34 days in 2013. The phenology and progression of cone development also varied between 2012 and 2013. In particular, cone development proceeded more rapidly from stage III to stage IV during 2012 versus 2013.

## Discussion

Both greenhouse and field experiments indicated that hop cones developed partial resistance to powdery mildew with maturity. This has important implications for targeting management practices to periods of greatest host susceptibility, as demonstrated in other pathosystems (e.g. [19,30,31]).

Results from detached tissue assays may be confounded by the artificial nature of the system. The detachment of cone tissues may affect the maturation process and impact still unknown factors involved in the development of ontogenic resistance. These potential artifacts were minimized in field studies that simulated more natural growing conditions. The field experiments demonstrated that the most substantial reductions in cone color, dry matter content of cones, and alpha acids were associated with infection during stage I and II of cone development. This provides some clarification to questions raised previously about damage to hop cones by powdery mildew [5,7]. Impacts of powdery mildew on development of bittering acids appears strongly linked to the timing of infection. Previous studies have indicated that development of alpha acids is sensitive to temperature during flowering [32], suggesting that flowering is a critical period for resin gland development and synthesis of bittering acids. In the current study, infection during or just after bloom (stage I) was deleterious to alpha acids content of cones, but later infection had minimal impact on alpha acids. Similarly, Gent et al. [7] did not observe an association between the incidence of cones with powdery mildew or duration of fungicide applications after bloom and the alpha acids content of cones at harvest. In light of the present findings, previous studies that found no evidence for an affect of powdery mildew on alpha acids content on cones were likely influenced by conditions that limited the severity of powdery mildew at bloom.

The present study also provides a biological explanation for the finding of Nelson et al. [14] that the majority of disease control on cones may be associated with one or two fungicide applications centered on stage II of cone development. This response can be explained, in part, by development of partial ontogenic resistance, which is most notably expressed in bracteoles and bracts as cones develop beyond stages II and III, respectively.

Development of partial ontogenic resistance was associated with multiple effects, including reduced germination of conidia of the fungus, frequency of penetration of the host, and fecundity. Age-related resistance is common in plants and may operate against a specific pathogen, or race, or may be broad spectrum [33]. The mechanisms underlying age-related resistance are not entirely clear but in some pathosystems age-related resistance is associated with expression of a specific resistance gene, activation of transduction pathways, and/or increased expression of genes involved with host cell wall reinforcement and pathogen defense [33]. In the few systems where ontogenic resistance has been characterized in powdery mildew fungi, the cumulative effect of ontogenic resistance on the pathogen is manifested in processes that occur during and after host penetration. Ontogenically resistant tissues result in reduced frequency of penetration of the host, inhibition of hyphal development, lengthening of the latent period, and suppression of sporulation of the fungus [31,34]. These processes also appear to occur in hop cones, although we also find evidence that germination of conidia is reduced on more mature bracts. While the precise underlying mechanisms remain to be elucidated, the processes converge to produce a phenotype of overall reduced susceptibility to powdery mildew.

Nelson et al. [16] found that use of the fungicide quinoxyfen during a three week period in late July and early August yielded the best control of powdery mildew. The reason that this period is so long may be related to the presence of the cone stages most susceptible to powdery mildew being present over a relatively extended period of time. Bloom and cone development is highly asynchronous in the Pacific Northwest. This may complicate disease management efforts because the cone developmental stages most at risk from powdery mildew may be present for up to four weeks or longer. In grapevine protracted bloom has been associated with mild winter temperatures [35]. The impact of climactic factors on plant phenology has been described at the whole plant level in hop and for the chemical constituents of cones [36,37], but has yet to be investigated in detail for cone development. The commencement of bloom in hop is related to day length, a minimum number of nodes, and pollination [4,38,39], and these factors vary among cultivars. Factors associated with synchrony of bloom in hop are largely unknown, but seem likely to include interactions of cultivar, temperature, and horticultural factors such as the timing and uniformity of training.

Further, the specific efficacy of quinoxyfen found by Nelson et al. [16] may be related to protection of the abaxial surfaces of young, developing bracts. *P*. *macularis* was able to penetrate the abaxial surface of bracts at nearly twice the frequency as that of the adaxial surface and, in turn, developed more extensive hyphal microcolonies. Attaining fungicide coverage and protection of the abaxial surface of bracts is exceptionally difficult given the complex surface area of hop cones. The critical importance of applying efficacious fungicides during stage II of cone development may be explained in part by coverage of juvenile bracts and bracteoles before cone maturation leads to tightly overlapping bracts and bracteoles less penetrable to fungicide sprays.

The timing and severity of cone infection by *P*. *macularis* was linked to the frequency of isolation of a suite of saprophytic and opportunistic fungi. A similar phenomenon was described by Gadoury et al.[9] on grape clusters when inoculated with *E*. *necator* just prior to the development of ontogenic resistance. Although the organisms involved differed between hop and grape, we also found a clear role of powdery mildew in the development of a complex of weak pathogens on hop cones. Of particular note is *A*. *alternata*, an organism (or species complex) ubiquitous in soil and crop debris [40]. *A*. *alternata* may cause superficial damage to hop cones predisposed to infection from damage from heavy rains, wind, or physical abrasion [10,11]. In the Pacific Northwest, particularly Washington State where powdery mildew tends to be most severe on cones [28], fungicide applications have sometimes been applied late in the season when cone discoloration first becomes apparent in an effort to minimize what has been thought to be Alternaria cone disorder. These efforts seem entirely misguided in light of the findings here. Occurrence of *A*. *alternata* and other opportunistic organisms appear intimately associated with damage to preformed host barriers, and an important cause of such damage is prior attack by *P*. *macularis*. Therefore, appropriate management of powdery mildew is likely to reduce the occurrence of, and obviate later management efforts for, a complex of minor fungal diseases.

In the western U.S., powdery mildew is the disease of most concern during cone development. Thus, it is readily feasible to intensify management efforts to target the most critical stages of cone susceptibility to powdery mildew. The experiments with limited fungicide applications demonstrate the concept that the efficiency of a fungicide program depends to a large extent on the efficacy of powdery mildew disease control during a relatively short period of cone development. Ontogenic resistance also is known to occur in hop leaves [22] and the amount of juvenile leaf tissue is associated with the number of conidia produced and epidemic velocity [5]. Development of hop leaves is greatly reduced or ceases near bloom as plants transition from vegetative to reproductive development [4]. This dynamic of plant development and juvenile susceptibility of leaves leads to a consistent and progressive decline in the incidence of leaves with powdery mildew after bloom, even in the absence of disease control measures. This provides further motivation for targeting late season disease control to cones and selecting fungicides that are most efficacious against the cone phase of the disease.

However, we caution it may be ill-advised to adopt a limited spray program in commercial situations with highly susceptible cultivars because early intervention is critical to keep disease pressure on leaves at manageable levels [22,41]. The importance of disease control in the current season to overwintering of the pathogen also is unclear, and uncontained disease outbreaks could lead to greater perennation of *P*. *macularis*. Rather, the important conception here is that the juvenile stages of cone development are critical periods for disease control, and fungicide applications and other management actions are most essential during this period.

The careful deployment of a limited number of fungicide applications for maximum benefit is especially crucial to sustainable management of powdery mildews. In nearly all major crops where fungicides are the primary method of disease suppression, powdery mildew fungi have shown a marked propensity to develop resistance to most of the major fungicide groups. Hop cultivars grown in the Pacific Northwestern United States range in susceptibility to powdery mildew but at present there are few cultivars that are completely resistant to the disease [42]. Thus, powdery mildew must be controlled through cultural practices and the deployment of fungicides, on average 6 or more applications per year on susceptible cultivars [43]. That fungicide resistance has not yet been manifested in *P*. *macularis* should highlight the importance of preserving the efficacy of the remaining effective compounds through their judicious deployment, taking advantage when possible of natural expressions of ontogenic resistance in hop cones.

The value of fungicide applications beyond stage II of cone development requires careful consideration. Since no developmental stage of cones was immune, the incidence of cones with powdery mildew continues to increase over time (as noted in Fig. 5). These later infections often are manifested as diffuse colonies, most prominently on bracts, which are associated with epidermal necrosis and a general discoloration of cones [44]. Such infections may lead to increased colonization by secondary organisms, as demonstrated herein. As observed in the field experiments, these late season attacks by *P*. *macularis* may be obfuscated by the generally longer latent period of the fungus and its diminished sporulation. However, the consequence of such infections may be important for both yield and quality factors dependant on aspects such as intended use of the harvested hops, harvest date, severity of disease, and perhaps weather dynamics [7,44,45]. These factors are situation-dependent and, therefore, in some circumstances additional fungicide applications and other disease management efforts may be warranted to maximize cone yield and quality.

In practice, targeting fungicide and management strategies around periods of enhanced juvenile susceptibility may be confounded by the degree of bloom asynchrony, which may vary from season-to-season due to environment and horticultural practices. Further research is needed to understand factors that underlie development of ontogenic resistance, as well as understand production practices that may contribute to asynchronous bloom. With this knowledge, disease hazard warnings and late season disease management could be further refined.

### Disclaimer

The use of trade, firm, or corporation names in this publication is for the information and convenience of the reader. Such use does not constitute an official endorsement or approval by the United States Department of Agriculture or the Agricultural Research Service of any product or service to the exclusion of others that may be suitable.
